# Prevalence and correlates of low serum calcium in late pregnancy: A cross sectional study in the Nkongsamba Regional Hospital; Littoral Region of Cameroon

**DOI:** 10.1371/journal.pone.0224855

**Published:** 2019-11-07

**Authors:** Atem Bethel Ajong, Bruno Kenfack, Innocent Mbulli Ali, Martin Ndinakie Yakum, Phelix Bruno Telefo

**Affiliations:** 1 Kekem District Hospital, Kekem, West Region, Cameroon; 2 Department of Biochemistry, University of Dschang, Dschang, West Region, Cameroon; 3 Department of Obstetrics / Gynaecology and Maternal Health, Faculty of Medicine and Pharmaceutical Sciences, University of Dschang, Dschang, West Region, Cameroon; 4 Medecins Sans Frontieres-Spain (MSF-OCBA), Maiduguri, Nigeria; University of Mississippi Medical Center, UNITED STATES

## Abstract

**Introduction:**

Women from low and middle income countries are generally more likely to have sub-optimal calcium intake. The objective of this study was to assess serum calcium disorders and correlates in late pregnancy.

**Methods:**

We conducted from December 2018 to April 2019, a cross-sectional hospital-based study targeting pregnant women in late pregnancy in the Nkongsamba Regional Hospital. Data were collected by measurement of parameters (weight, height, blood pressure and foetal birthweight), administration of a semi-structured questionnaire and analysis of blood samples collected from each participant. Absorption spectrophotometry was used to measure serum calcium and albumin concentrations and corrected serum calcium calculated from the Payne’s equation. With a statistical significant threshold set at p-value = 0.05, the odds ratio was used as a measure of the strength of association between hypocalcaemia and maternofoetal variables.

**Results:**

We enrolled a total of 354 consenting participants with a mean age of 27.41±5.84 years. The prevalence of hypocalcaemia in late pregnancy was 58.76 [53.42–63.90]%. The rate of calcium supplementation in pregnancy was 57.63[52.28–62.80]% with a mean duration of supplementation of 3.69±1.47 months. When controlled for marital status, age, level of education, and gestational age at delivery, pregnant women with systolic blood pressures below 130 mmHg were significantly less likely to have hypocalcaemia than their counterparts with higher systolic blood pressures (Adjusted Odds Ratio = 0.41[0.18–0.89], p-value = 0.020). No statistically significant associations were found between diastolic blood pressure, body mass index, foetal birth weight and hypocalcaemia.

**Conclusion:**

Hypocalcaemia in late pregnancy is highly prevalent (59%) among women accessing reproductive services at the Nkongsamba Regional Hospital. There is also a wide gap in calcium supplementation compared to World Health Organization recommendations. Hypocalcaemia is significantly associated to higher systolic blood pressure in pregnancy. Systematic calcium supplementation and consumption of high calcium containing locally available meals should be encouraged.

## Introduction

The state of pregnancy is known to increase calcium demands, which need to be met. Even though calcium homeostasis is finely regulated by a complex mechanism coordinated by a variety of hormones, total serum calcium levels have been described to be consistently low among pregnant women[1]. The serum levels of ionized calcium in recent literature have however been described to remain sensibly unperturbed during pregnancy[2–6]. Even though physiologically regulated in pregnancy, studies in India [7] and Algeria [8] have reported a very high prevalence of hypocalcaemia in pregnancy (66.4% and 70.55% respectively).

Circulating calcium in plasma occurs in three forms: free ions (51%), protein-bound complexes (40%), and ionic complexes (9%). Normal total serum calcium concentration ranges from 8.8 to 10.4 mg/dl (2.2 to 2.6 mM) in healthy subjects [9]. This value however is usually subjected to significant variations depending on factors like parathyroid hormone and calcitonin secretion, dietary intake and other conditions like osteoporosis [3]. Calcium can be gotten directly from food sources and dietary supplements. Dietary calcium intake and absorption are essential in providing sufficient calcium to maintain healthy body stores.

Substantial evidence has associated hypocalcaemia in pregnancy to several life-threatening morbidities. Maternal hypocalcaemia has been associated with high blood pressure, pregnancy induced hypertension [10], pre-eclampsia [11–13] and increased serum lead levels [14]. It has also been associated with foetal morbidities like neonatal low bone mass [15], poor foetal growth [16] and increased risk of small for gestational age [17].

A major source of calcium is in dairy products like milk, yogurt, and cheese which constitute rich sources of calcium, providing the major share of calcium from foods in the general diet [9, 18–20]. The consumption of these products is however not consistent in our setting especially in the semi-urban settings. Calcium supplementation during pregnancy and antenatal care in Cameroon is not systematic and the systematic antenatal package of work-ups does not consider measurements of serum calcium levels at different trimesters for subsequent correction. The Prescription of calcium supplements for pregnant women in our milieu is practitioner-dependent.

To the best of our knowledge, multiple studies have been conducted in the developed world to describe serum calcium disorders in pregnancy but these studies are however sparse in our setting. The burden of hypocalcaemia in late pregnancy; period during which foetal demands are maximum has not been studied in semi urban Cameroun (a population with a different socio-cultural and nutritional status). According to the World Health Organization, women who chronically take in suboptimal amounts of calcium (<500 mg/day) may have an increased risk of hypocalcaemia and bone loss during pregnancy [21].

According to two recent systematic reviews, low and middle income countries are more likely to have significantly lower calcium intake compared to countries of the developed world [22, 23]. Women in our setting are therefore likely to have gone into pregnancy with already suboptimal calcium intake and low serum calcium. We therefore designed this study to estimate the prevalence of hypocalcaemia in late pregnancy among a sample of women accessing reproductive health services at the Nkongsamba Regional Hospital (NRH), determine their rate of calcium supplementation during pregnancy, and describe the association between hypocalcaemia in late pregnancy, and some maternofoetal variables (systolic and diastolic blood pressure, body mass index, cramps and foetal birthweight).

## Materials and methods

### Study design

A cross-sectional hospital-based study was conducted from December 2018 to April 2019 in the maternity of the NRH targeting apparently healthy pregnant women in their third trimester of pregnancy. The study consecutively enrolled all eligible and consenting pregnant women received in the hospital from December 2018 to April 2019 for antenatal consultations. The outcome of this study was corrected serum calcium levels. Data collected were keyed with Epi-info version 7.2.2.16 and data analysis was first of all descriptive to measure the prevalence of hypocalcaemia and then analytic with regression technics to assess factors associated with hypocalcaemia.

### Study site

Data and sample collection were carried out at the maternity of the NRH. NRH is the major referral hospital of the Moungo division and particularly the Nkongsamba Health District (in the Littoral Region of Cameroon). This hospital serves patients from most of the Moungo division and according to its maternity statistics, it is known to conduct on average 100 new antenatal consultations and about 100–150 deliveries monthly. The laboratory samples collected were immediately transported to the biochemistry laboratory of the Bethanie group of laboratories (Moungo Branch). This is a specialised multi-purpose clinical laboratory that mostly serves the patients of the Moungo division. The Moungo branch of this laboratory is located about two kilometres away from the NRH.

### Study population

The study targeted all pregnant women in their third trimester of pregnancy. Eligible participants included all pregnant women received at the maternity of the NRH for routine antenatal consultations in the late third trimester (greater than or equal to 37 weeks of gestation). Our study however did not include participants with chronic pathologies like diabetes and hypertension or known carriers of parathyroid disorders or vitamin D deficiency (and its causes like chronic liver disease, kidney disease or evident malnutrition). These participants were excluded based on verification of their medical records, their declarations, and clinical evaluation. Participants with intra-uterine foetal demise or multiple gestations were also not included in the study.

### Sampling and sample size

Our sampling was consecutive and purposive in nature. The minimum required sample size was estimated to 352 participants using the following parameters: The expected proportion of women with hypocalcaemia in pregnancy (70.55% from a recent Algerian study) [8], the absolute precision required on either sides of the proportion, a threshold of error at 5%, the confidence interval at 95% and a non-response rate of 10%.

### Procedure of implementation and data collection

When the protocol, the questionnaire and informed consent form were ready, the data collection tools were pretested and validated on a sample of 20 pregnant women in the Kekem District Hospital (West Region of Cameroon). Administrative authorizations and ethical clearance were obtained prior to data collection and blood sampling. All these were then submitted to the national ethical committee for ethical evaluation and approval. Two nurses were recruited and trained (two training sessions of three hours each) on the consenting, data and sample collection procedures.

To each participant was fully presented the information notice of study. Willing participants were made to sign informed consent or assent forms where applicable. A semi-structured questionnaire was then administered face to face by interview. The blood pressure, the weight and height of the participants were measured using an aneroid sphygmomanometer, a digital weighing scale and a standing height measuring device respectively. The brachial blood pressure was measured following about 5–10 minutes of rest in a sitting position and entered into the questionnaire in millimeters of mercury. Blood pressure was measured twice in each participant in sitting position and the mean systolic and diastolic value registered on the questionnaire for analyses.

The weight of the participant was taken in a standing position using the digital weighing scale and included in the questionnaire in kilogrammes. The height of each participant was also measured in an erect position, using a graduated height measuring scale and registered in centimeters. Weight and height of participants in this study was measured with participants wearing light clothing with emptied pockets and without shoes. Body weight was measured to the nearest 0.1 kg on a digital weighing scale and height to the nearest 0.5 cm. BMI in this study was estimated as weight minus 1 kg to adjust for clothing, divided by height squared (kg/m2). Each pregnant woman included in the study was followed up till delivery and the birth weight of the baby taken using a baby digital weighing device. This weight was taken immediately after delivery before dressing up the baby.

Prior to measuring the blood pressure of the participants, 2ml each of venous blood was collected by well-trained nurses without squeezing of the vein using a vacuum blood collection needle into heparinised tubes. This was done after cleaning with a cotton swap imbibed in alcohol. Because of in-vitro pH changes that can affect serum calcium levels after sample collection, collected samples were centrifuged immediately and stored in the freezer at 4 degrees Celsius and transported in ice parks to the laboratory after every two hours. The heparinised plasma collected after centrifugation was used for calcium and albumin assays.

### Procedure of serum calcium and albumin assays

Our study adopted measurement of total plasma calcium by atomic absorption spectrophotometry which was corrected by concomitant measures of plasma albumin. CPC (O-Cresol Phtalein Complexone) method was used to measure total calcium concentration in heparinised plasma. In alkaline solution CPC reacts with calcium to form a dark-red coloured complex. The absorbance of this coloured complex measured at 570 nm is proportional to the amount of calcium in the specimen. The semi-automatic spectrophotometer KENZA MAX Biochemis Try of the manufacturer BIOLABO was used to measure calcium concentrations in heparinised plasma per 2019 BIOLABO standard operating procedure [24]. As concerns serum or plasma albumin, in buffered solution and at pH 4.2, bromocresol green (BCG) binds albumin to form a coloured compound whose absorbance, measured at 630 nm (620–640) is proportional to the albumin concentration in the specimen. The serum albumin concentration was measured using the same spectrophotometer with the bromocresol green reagent of BIOLABO as per the 2011 BIOLABO standard operating procedure [25].

Variations in serum albumin concentration have been described to significantly alter the concentration of total serum or plasma calcium [26]. A dozen of equations have been proposed for estimation of corrected plasma or serum calcium but the most widely used and accepted equation remains Payne’s equations; Corrected serum or plasma calcium (mmol/L) = Total serum or plasma calcium (mmol/L) + 0.02 [40 –albumin (g/L)] [27], routinely used in clinical practice to give an estimate of calcium concentration in patients with hypoalbuminemia. We therefore adopted this equation for estimates of our corrected serum calcium given that pregnant women are generally exposed to reduced serum albumin concentrations.

### Data analysis

Data from the questionnaire and blood assays were keyed into an epi-info version 7.2.2.16 predesigned data entry sheet and analysed. Any samples with albumin-corrected serum calcium of less than 8.5 mg/dl were considered to contain low serum calcium levels (hypocalcaemia). The BMI (a crude BMI calculated using the weight of the pregnant woman at the time of data collection) of the participants was categorised into BMI≥30kg/m^2^ and less. The systolic and diastolic blood pressure were categorised into higher or lower than 130 mmHg or 70 mmHg respectively. The weight of the baby at birth also was categorised into greater than, and less than 3000g.

Major statistical analyses included calculation of frequencies and their 95% confidence intervals for categorical variables (age-groups, marital status, level of education, frequency of hypocalcaemia, frequency of calcium supplementation), and means and medians for continuous variables where applicable (age, corrected calcaemia, duration of calcium supplementation). As concerns the prevalence of hypocalcaemia, participants’ corrected serum calcium levels were categorised into low or normal based on the above cited cut-off (8.5g/dl). The prevalence of serum hypocalcaemia was determined as the proportion of pregnant women at 3^rd^ trimester with a corrected serum calcium concentration less than 8.5g/dl. This estimation was calculated alongside its 95% confidence interval. The strength of association between serum calcium levels and covariates (systolic blood pressure, diastolic blood pressure, foetal birthweight, and body mass index) was measured using the odds ratio and their 95% confidence intervals by simple logistic regression (threshold of significance set at p-value less than 0.05). Some covariates from simple logistic regression were then controlled for marital status, age, level of education, and gestational age at delivery in a multiple logistic regression model with a threshold of significance set at p-value less than 0.05.

### Ethical consideration

Ethical approval for this study was obtained from the national ethics review board for studies on human subjects in Cameroon (Ethical clearance number: 2019/05/24/CE/CNERSH/SP). An information notice was introduced to each participant or their legal representatives during which he or she was well enlightened on the objectives of the study and the potential risk associated, leaving the participant to willingly accept to participate or withdraw from the study without any compensation for the participants nor any penalization for those that refused to participate. Willing participants signed an informed consent form before enrollment (an assent form was administered to minors and signed consent obtained from their legal representatives or guardians) and all data was analyzed anonymously.

## Results

### Socio-demographic and obstetric characteristics of the participants

A total of 402 women were contacted for the study, 374 met inclusion criteria and 20 refused to participate leaving us with 354 eligible participants. Table 1 summarises the socio-demographic and obstetric characteristics of the participants. The mean age of the participants was 27.41±5.84 years with an age range of 15–48 years (About 8 in every 10 women between 21–40 years old). Among these participants, about 7 in every 10 (35.31% married and 33.90% in a consensual union) were in a union and 30.23% single.

**Table 1:** Socio-demographic and obstetric characteristics of the study population.

Variable	Frequency	Percentage
** Age groups (years)**		
15–20	40	11.30
21–25	107	30.22
26–30	99	27.97
31–49	108	30.51
** Marital status**		
Married	125	35.31
Consensual or free union	120	33.90
Single	107	30.23
Divorced	02	0.56
** Occupation**		
Nothing	31	8.76
Student	103	29.10
Skilled	109	30.79
Unskilled	111	31.36
** Level of education**		
Never schooled	00	00.00
Primary	19	5.37
Secondary	280	79.10
Higher	55	15.54
** Number of pregnancies**		
First pregnancy	91	25.71
Two to three	156	44.07
Four to six	98	27.68
Above six	09	02.54
** Number of children alive**		
One	112	31.64
Two to three	156	46.61
Four to six	76	21.47
Above six	01	0.00
** Gestational age at delivery in weeks**		
37	61	17.23
38	84	23.73
39	79	22.32
40	85	24.01
41	27	07.63
42	18	05.08

All participants had attended at least primary education. About 9 in every 10 had acquired at least a secondary education (79.10% for secondary education and 15.54% for higher education). The population was therefore dominated by participants with secondary education as their highest level of education. At least 6 in every 10 participants had a job of one type or the other (62.15%). Up to 29.10% of our participants were students. Our study population had a mean total number of pregnancies of 3 (2.83±2.78) with 44.07% having a history of two to three pregnancies. At least 4 in every 10 (46.61%) had two to three living children. The mean gestational age at delivery was 38.96±1.93 weeks.

### The prevalence of hypocalcaemia in late pregnancy

The mean albumin-uncorrected (crude) and albumin-corrected calcaemia of the study population was 7.83±0.71 mg/dl and 8.47±0.66 mg/dl respectively (p-value<0.001). The prevalence of albumin-uncorrected and corrected hypocalcaemia (the standard prevalence of hypocalcaemia for our study) in late pregnancy was 85.88 [81.71–89.24]% and 58.76 [53.42–63.90]% respectively.

Table 2 below shows the variation of the corrected prevalence of hypocalcaemia with some maternal variables. The variation across age groups was not significant with peak prevalence (65.42%) between 21–25 years. The variation across the different number of pregnancy groups was mild with a peak prevalence (62.82%) recorded in women who reported two to three total pregnancies. Peak rates of hypocalcaemia were observed among students (65.05%), single/divorced participants (66.97%), and participants who had acquired just primary education (73.68). The variation of the prevalence of hypocalcaemia with socio-demographic characteristics like age, marital status, occupation, number of pregnancies, level of education was not statistically significant (p-value>0.05.

**Table 2:** Variation of the prevalence of hypocalcaemia with age groups, marital status, total number of pregnancies, level of education and occupation.

Variable	Frequency of hypocalcaemia (%)
Age group (in years)	
15–20 (n = 40)	22(55.00)
21–25 (n = 107)	70(65.42)
26–30 (n = 99)	56(56.57)
31–49 (n = 108)	59(54.63)
Marital Status	
Women in union (n = 245)	135(55.10)
Single/divorced (n = 107/2)	73(66.97)
Total number of pregnancies	
First pregnancy (n = 91)	52(57.14)
Two or three (n = 156)	98(62.82)
Four to six (n = 98)	53(54.08)
Above six (n = 9)	05(55.56)
Level of education	
Never schooled (n = 00)	NA
Primary (n = 19)	14(73.68)
Secondary (n = 280)	158(56.43)
Higher (n = 55)	36(65.45)
Occupation	
Skilled (n = 109)	59(54.13)
Unskilled (n = 111)	66(59.46)
Student (n = 103)	67(65.05)
Nothing (n = 31)	16(51.61)

NA: Not Applicable

### Rate of calcium supplementation in pregnancy

The rate of calcium supplementation in pregnancy among the study population was 57.63 [52.28–62.80]% with a mean duration of supplementation of 3.69±1.47 months. Even though not statistically significant, women who had taken calcium supplementation for more than 4 months during their pregnancy were less likely to have hypocalcaemia compared to their counterparts who had taken calcium for less than three months (OR = 0.726 [0.38–1.39], p-value = 0.335).

A little less than two-thirds of the study population (62.15%) reported to have experienced an increased frequency of cramps during their pregnancy. There was a statistically significant association between women who experienced increased frequency of cramps in pregnancy and calcium supplementation. Pregnant women with increased frequency of cramps were 1.65 times more likely to be on calcium supplementation compared to their counterparts who experienced no changes in the frequency of cramps (OR = 1.65 [1.07–2.55], p-value = 0.024).

The prevalence of hypocalcaemia among calcium supplemented and non-calcium supplemented women was 61.27 [54.00–68.00]% and 55.33 [47.01–63.45]% respectively. Pregnant women on calcium supplementation were more likely to have low serum calcium levels during their pregnancy compared to their counterparts with no supplementation. This association was however not statistically significant (OR = 1.27 [0.83–1.96], p-value = 0.262).

### Association between hypocalcaemia and maternofoetal variables

Table 3 below shows analysis results of the association between hypocalcaemia and materno-foetal variables following simple logistic regression (results for multiple logistic regressions are not shown on the table).

**Table 3:** Determination of the association between hypocalcaemia and some maternal and foetal variables.

	HYPOCALCAEMIA		INFERENTIAL STATISTICS	
	YES	No	OR[95%CI]	p-value
**Systolic blood pressure less than 130 mmHg**				
Yes	178	136	0.44 [0.21–0.92]	0.030*
No	30	10		
Total	208	146		
**Diastolic blood pressure less than 70mmHg**				
Yes	121	93	0.79 [0.51–1.23]	0.296
No	87	53		
Total	208	146		
**BMI below 30 kg/m**^**2**^				
Yes	160	103	1.39 [0.86–2.25]	0.178
No	48	43		
Total	208	146		
**FBW less than 3000g**				
Yes	82	52	1.18 [0.76–1.82]	0.468
No	126	94		
Total	208	146		

OR: Odds Ratio, 95%CI: 95% Confidence Interval, BMI: Body Mass Index, FBW: Foetal Birthweight

*statistically significant (p-value<0.05).

There was an inverse association between systolic blood pressure and serum calcium. Pregnant women with systolic blood pressures below 130 mmHg were significantly less likely to have hypocalcaemia than their counterparts with higher systolic blood pressures (OR = .0.44 [0.21–0.92], p-value = 0.030). When controlled for age, level of education, marital status and gestational age at delivery in a multiple logistic regression model, the association between systolic blood pressure and calcaemia remained statistically significant (Adjusted Odds Ratio = 0.41 [0.18–0.89], p-value = 0.020).

Decreasing diastolic blood pressure was insignificantly associated to decreasing likelihood of hypocalcaemia in pregnancy following simple logistic regression (p-value = 0.296). This association remained statistically insignificant when adjusted for age, marital status, level of education and gestational age at delivery in a multiple logistic regression model (Adjusted Odds Ratio = 0.73 [0.46–1.15], p-value = 0.178).

No statistically significant association was found between body mass index and hypocalcaemia (p-value = 0.178) following simple logistic regression. When controlled for marital status, age, level of education, and gestational age at delivery, this association remained statistically insignificant (Adjusted Odds Ratio = 1.40 [0.83–2.34], p-value = 0.210).

Following simple logistic regression analysis, foetal birth weight (p-value = 0.468) had no significant association with the likelihood of hypocalcaemia.

## Discussion

We conducted a hospital-based cross-sectional study targeting women in late pregnancy. We studied albumin-corrected serum calcium levels in a sample of 354 women in late pregnancy. This study presents preliminary but vital information on the burden of hypocalcaemia in the third trimester of pregnancy in a semi-urban setting in Cameroon.

Our study recorded a prevalence of hypocalcaemia in late pregnancy of 58.76%. This prevalence was even significantly higher when serum calcium values were uncorrected (85.88%) for the albumin changes in pregnancy. The perceived burden of hypocalcaemia in pregnancy is significantly different when albumin-corrected values are considered. Our findings reported a crude hypocalcaemia prevalence of 85% which when we corrected measured calcium levels for albumin changes in pregnancy, the prevalence significantly dropped to 59%. This implies that compared to the prevalence of hypocalcaemia, up to 27.12% (85.88%-58.76%) of these pregnant women with hypocalcaemia in our study population would be erroneously classified. According to our prevalence of hypocalcaemia, about 6 in every 10 pregnant women in their third trimester of pregnancy had inadequate serum calcium levels. Our finding on the prevalence of hypocalcaemia in late pregnancy shows that this metabolic imbalance is highly prevalent among pregnant women in this setting.

During the last trimester, calcium actively crosses the placenta from the mother to the foetus [28]. Significant umbilical arterio-venous differences have been reported as far as total calcium concentrations are concerned, and this differences reflect variations in the protein-bound form only [28]. Calcium is required in its maximal concentrations in the third trimester for foetal bone formation and consolidation; an explanation for the significantly high trans-placental transport of calcium in the third trimester [6, 29].

A similar study was conducted in Algeria targeting women in their third trimester and their babies. The authors reported a relatively higher prevalence (70.55%) of hypocalcaemia among pregnant women in their third trimester of pregnancy [8]. The prevalence of hypocalcaemia in our study (58.76%) even though seemingly lower is not very different from Benali and Demmouche’s findings [8]. Despite the fact that they considered a lower cut-off point (less than 8.0 mg/dl) to define hypocalcaemia among their pregnant women, they did not concomitantly normalise the measured calcium concentrations by serum albumin. Similarly, a very high prevalence of hypocalcaemia in pregnancy (66.4%) was reported among pregnant women in their third trimester in India [7]. They however still did not do concomitant measurements of serum albumin concentrations to correct their calcium concentrations. If any slight discrepancies persist between the different rates of hypocalcaemia registered in these three studies, methodological and nutritional differences between the different populations can explain them. Nutritional habits vary significantly across the different study populations given that the three studies were conducted in three different countries with significantly different sociocultural and economic status.

Poor nutrition significantly affects maternal and infant morbi-mortality. Calcium supplementation reduces adverse gestational outcomes [21], in particular, by decreasing the risk of developing hypertensive disorders during pregnancy, which are associated with a high maternofoetal morbi-mortality [30]. Despite its clear advantages, the rate of calcium supplementation among our study population (57.63%) was very low compared to WHO recommendations for a low income country (with increased likelihood of chronic low calcium intake in meals) [21]. This implies that over 40% of these women in a setting where women were likely to have gone into pregnancy with sub-optimal calcium intake went through their pregnancy without any calcium supplementation. Our findings concord with those in a Benin cross sectional survey on calcium uptake, which indicate that over 90% of pregnant women in Southern Benin had low calcium intake during their pregnancy [31].

The WHO recommends that women be put on calcium supplementation from their booking visits and the daily dose (1.5-2g) divided into two or three [21]. The mean duration of calcium supplementation in pregnancy in our study was about 4 months significantly lower to meet maternofoetal calcium demands. This could be explained by the fact that most women in our milieu book late for antenatal care. Even though not statistically significant, women who had taken calcium supplementation for more than 4 months during their pregnancy were less likely to have hypocalcaemia compared to their counterparts who had taken calcium for less than three months (OR = 0.72[0.38–1.39], p-value = 0.335). This is an indication that “the duration of supplementation might have an influence on likelihood of hypocalcaemia if fully exploited”.

Women on calcium supplementation may not be able to meet normal serum calcium levels because of a series of prescription errors and difficulties associated to our common food sources in this milieu. Given that intestinal calcium absorption doubles as early as from 12 weeks of pregnancy, calcium supplementation should be started as early as possible in pregnancy. It should be noted that not all calcium consumed is absorbed. Negative interactions between iron and calcium supplements in pregnancy have been described. Therefore, it is advised that the two nutrients be taken preferably several hours apart rather than concomitantly [21].

Pregnant women with increased frequency of cramps were 1.65 times more likely to be on calcium supplementation compared to their counterparts who experienced no changes in the frequency of cramps (p-value = 0.024). This is suggestive of the fact that calcium supplementation in pregnancy in this milieu might be dependent on the presence or absence of symptoms or is only intensified when patients present with cramps. This is however not a correct approach as up to 38.46% of participants with hypocalcaemia in pregnancy experienced no increase in the frequency of cramps. Current evidence does not suggest any statistically significant relation between calcium supplementation and decreased frequency of cramps in pregnancy [32]. According to findings by Zhou et *al* [32], the evidence that calcium reduces cramp is weak and seems to depend on placebo effect. The prescription of calcium supplementation is therefore not supposed to be based on particular symptoms in pregnancy but should follow recommendations.

Targeted dietary counselling of pregnant women should promote adequate calcium intake through locally available, calcium-rich foods associated to supplementation in our context. Studies to measure the calcium content of most of the cooked locally available meals and some designed to identify common calcium absorption inhibitors are indispensable for meeting calcium supplementation targets in this milieu. In addition, medical personnel need to be awakened to the need of calcium supplementation in pregnancy and the population adequately sensitized on it.

Pregnant women with systolic blood pressures below 130 mmHg were significantly less likely to have hypocalcaemia than their counterparts with higher systolic blood pressures (Adjusted Odds Ratio = 0.41[0.18–0.89], p-value = 0.020). In other words, there was an inverse relationship between systolic blood pressure values and serum calcium levels of the participants. Our findings on systolic blood pressure therefore are in line with WHO guidelines which recommend calcium supplementation as a means of preventing hypertensive disorders in pregnancy [21]. Hypertension has been estimated to complicate 5% of all pregnancies and 11% of first pregnancies. Half the women with hypertension present with pre-eclampsia. Hypertensive disorders account for up to 40 000 maternal deaths annually (they account for 14% of all maternal deaths) [30]. For this reason, strategies to reduce the risk of hypertensive disorders of pregnancy have received considerable attention.

A substantial load of evidence suggests that calcium supplementation and therefore all efforts to avoid hypocalcaemia can significantly reduce the risk of hypertensive disorders in pregnancy [11, 13, 33]. According to a systematic review by Imdad *et al*, calcium supplementation during pregnancy can reduce risk of pre-eclampsia by 52% and that of severe pre-eclampsia by 25%. They recorded a significant reduction for risk of maternal mortality/severe morbidity (RR 0.80 [95% CI 0.65, 0.97]) (12).

Possible mechanisms by which calcium might reduce pre-eclampsia include inhibition endothelial damage, underlying the development of preeclampsia or by just preventing the manifestation of pre-eclampsia by reducing blood pressure [13, 34]. Calcium supplementation during pregnancy for women with deficient dietary calcium intake is associated with significant benefits for individual women. However, a public health policy of calcium supplementation during pregnancy is unlikely to have a major impact on the incidence of pre-eclampsia in our milieu if not coupled to increased sensitization of the population to start antenatal care on time. We therefore suggest that future research be directed towards detection of strategies to improve calcium intake at a population level.

Decreasing diastolic blood pressure was insignificantly associated to decreasing likelihood of hypocalcaemia in pregnancy (p-value = 0.296). This association remained statistically insignificant when adjusted for age, marital status, level of education and gestational age at delivery in a multiple logistic regression model (Adjusted Odds Ratio = 0.73[0.46–1.15], p-value = 0.178). Even not significant statistically, the direction was very similar to our findings on systolic blood pressure. Relatively higher diastolic blood pressures were associated to hypocalcaemia.

Following simple logistic regression analysis, foetal birth weight (p-value = 0.468) had no significant association with the likelihood of hypocalcaemia. Even though not statistically significant, babies with birth weights less than 3000g were more likely to be issued from mothers with hypocalcaemia. This result suggests that there “might be a relationship between hypocalcaemia and foetal birth weight”. Our sample size calculated on the basis of a predominantly prevalence study, might not have been large enough to bring out this association (given the likelihood of the power of the study being small). Similar findings were however established between hypocalcaemia and FBW among a sample of 900 women in Algeria [12]. Seemingly contrasting findings were reported with a significantly higher sample compared to ours (1116 participants) in a prospective cohort study. According to their findings, a very small (weak) but significant association was found between very low calcium intake and birthweight [16].

Though not a statistically significant association, women with BMI less than 30kg/m^2^ were 1.4 times more likely to have low serum calcium levels compared to their counterparts with higher BMI. Changes in human microbiome and the feeding habits among these women could explain the differences in the weight and therefore BMI. Several prebiotics that reach the lower intestine have been described to alter the gut microbiome that is thought to enhance fermentation of the fibers to produce short-chain fatty acids. These changes are positively associated with increases in fractional calcium absorption in adolescents and with increases in measures of bone density and strength in animal studies [35]. According to a study conducted on obesity and calcium absorption, metabolic status influences calcium absorption such that severe obesity is associated with higher calcium absorption [36].

Hypocalcaemia in pregnancy as shown by our results, supported by existing findings in literature constitutes a serious and relatively very frequent disorder in pregnancy. The deficits in calcium supplementation and a probable inability to meet up calcium intake with locally available meals remain the most probable explanations for this high rate of hypocalcaemia. Hypocalcaemia is associated with increasing blood pressures in pregnancy and therefore hypertensive diseases in pregnancy which are generally connected with significant maternofoetal morbi-mortality. Calcium supplementation and counselling to encourage increased consumption of high calcium-containing locally available meals is indispensable in the fight against this metabolic imbalance and its deadly complications.

The data herein should however be interpreted with care. Although the number of participants considered in our survey may not be large enough to provide adequate power to detect differences, our survey provides true levels of calcaemia in late pregnancy, which has helped us to generate hypothesis for our future study. Our survey could not respond to a number of research worries in this milieu and field: we could not in full explore the feeding habits as well as measure the calcium contents of the different consumed foods among these women, the different forms of calcium supplements taken and the posology (and whether the supplements are taken in combination with some calcium absorption inhibitors); data which could help explain the high prevalence of hypocalcaemia. Barriers to calcium supplementation in this population were not evaluated. Our study focused principally in the late third trimester; certainly, the prevalence differs with the pregnancy age-range considered and among young non pregnant women of child bearing age. Also, we did not extend our study to measure and detect other electrolyte disorders in pregnancy like is the case with magnesium. This and many others constitute future perspectives.

Ideally, the ionised serum calcium levels which constitute the physiologically active fraction of serum calcium should be measured but because of the unavailability of this diagnostic tool (not used in routine laboratories in Cameroon), our study measured total serum calcium concentrations which were then albumin-corrected. Future studies should use sophisticated calcium analysers capable of measuring ionised calcium levels.

The validity of Payne’s equation to correct total serum calcium has not been studied and reported in pregnancy. We however did not find any alternative valid formula which could be adopted in pregnancy. In addition, Payne’s equation was principally derived for changes in albumin concentrations generally found in pregnancy (reduced serum albumin concentrations) and is generally accepted in clinical practice as a standard formula to evaluate corrected serum calcium in healthy patients.

Despite the above mentioned limits, our findings provide baseline information on this metabolic imbalance in Cameroon. This can already help design interventions to fight this metabolic imbalance in this context and can go a long way in helping prevent low-calcium associated maternal and foetal morbi-mortality in this setting.

## Conclusion

Based on our research findings, inadequate serum calcium levels in late pregnancy are highly prevalent (59%) among pregnant women accessing reproductive health services in the NRH. We additionally observed a wide gap in calcium supplementation with respect to WHO recommendations. The deficits on calcium supplementation in this context are significantly high serving as a call for action. Moreover, our study reinforces evidence on the relationship between hypocalcaemia and hypertensive diseases in pregnancy. Higher systolic blood pressures were found to be significantly associated to hypocalcaemia. It was however found that the association between third trimester hypocalcaemia and other variables like diastolic blood pressure, BMI, and FBW were statistically not significant. Globally, morbidities associated to low serum calcium in pregnancy could be significantly limited by encouraging systematic calcium supplementation in pregnancy and increased consumption of high calcium-containing locally available meals. Future research perspectives in this population should evaluate the burden of this serum disorder just before pregnancy, and in the other trimesters in pregnancy, screening of the feeding habits of this population and detecting if any, the consumption of substances interfering with intestinal calcium absorption among others.

✓ Dr MOBY Edouard Hervé; Director of the Nkongsamba Regional Hospital for accepting to habour this study in your hospital.
✓ To the entire staff of the maternity unit for their constant support and guidance during the data collection procedure.
✓ To the Bethanie Group of laboratories and particularly to the staff of its Moungo branch for their devoted attentions towards sample analysis.
✓ All the pregnant women of Nkongsamba Regional Hospital who accepted to participate in this research.

## Supporting information

S1 Data Data base on serum calcium disorders in late pregnancy among women in the Nkongsamba Regional Hospital. (MDB)

## Abbreviations

AOR: Adjusted Odds Ratio
BCG: Bromocresol Green
BMI: Body Mass Index
CI: Confidence Interval
CPC: O-Cresol Phthalein Complexone
FBW: Foetal Birth Weight
OR: Odds Ratio
PTH: Parathyroid Hormone
RR: Relative Risk
NRH: Nkongsamba Regional Hospital
WHO: World Health Organization
